# Identification of Pediatric Bacterial Gastroenteritis From Blood Counts and Interviews Based on Machine Learning

**DOI:** 10.7759/cureus.43644

**Published:** 2023-08-17

**Authors:** Yoshifumi Miyagi

**Affiliations:** 1 Department of Pediatrics, Haibara General Hospital, Shizuoka, JPN

**Keywords:** white blood cells, blood eosinophil count, platelet to lymphocyte ratio, blood count, decision tree, lasso, machine leaning, gastroenteritis in children

## Abstract

Introduction: Differentiating between bacterial and viral gastroenteritis is crucial in pediatric enteritis practice. Our objective was to use machine learning (ML) to identify acute gastroenteritis (AG) caused by bacteria based on blood cell counts and interview findings.

Methods: ML was performed using a decision tree classifier based on data from previously published papers. We included 164 children between one and 108 months diagnosed with gastroenteritis, with 112 having bacterial AG and 52 having viral AG as subjects and controls. Feature selection was performed using least absolute shrinkage and selection operator (LASSO), and the classifier's performance was evaluated by five-fold cross-validation. Additionally, we presented a tree diagram of the decision tree classifier as a flowchart for practical applications.

Results: The area under curve (AUC) was 0.80, indicating a moderate model. Three important features in this model were platelet-lymphocyte ratio, eosinophil count, and leukocyte count.

Conclusions: In conclusion, this study demonstrates that bacterial AG can be estimated from blood cell counts with moderate accuracy. These findings may be valuable in narrowing down bacterial AG in children with gastrointestinal symptoms.

## Introduction

Acute gastroenteritis (AG) is a common illness usually caused by viral or bacterial infections of the gastrointestinal tract. The main clinical manifestations of AG include nausea, fever, and the sudden onset of vomiting and diarrhea, with or without abdominal pain [1]. However, differentiating between viral and bacterial gastroenteritis based solely on clinical symptoms is challenging [2]. Both viral and bacterial gastroenteritis can present with sudden onset of vomiting, watery diarrhea, low-grade fever, and abdominal pain, making it difficult to distinguish between the two [3]. Therefore, further evaluation of the differences between viral and bacterial gastroenteritis holds significant clinical importance.

Additionally, bacterial gastroenteritis is less common compared to viral gastroenteritis [4]. In cases of severe bacterial enteritis, antimicrobial agents may be necessary [5]. Differentiating between bacterial and viral enteritis is crucial in the treatment of acute gastroenteritis (AG) in children [6]. While the gold standard for diagnosing bacterial enteritis is bacterial culture [7], this process is time-consuming and requires specialized equipment [8]. Stool polymerase chain reaction is a useful current molecular biological technique, but it has initial set-up and cost issues [9]. Another disadvantage is the inability to distinguish between dead and viable bacteria, and results must be interpreted carefully according to the patient's condition [9]. Especially in less well-equipped settings, the determination of AG bacteria at the time of consultation relies on the findings of the pediatrician, but specific indicators are not clearly defined.

To address this, we employed machine learning (ML) to create a model that identifies bacterial AG using blood counts and interview data to identify significant features. Additionally, we utilized a decision tree classifier to construct a tree diagram that can be utilized during actual consultations.

## Materials and methods

Data collection

Ethics committee approval was not required because this study uses publicly available supplemental data from previously published articles. The study included children presenting to pediatric emergency departments with acute community-acquired infectious diarrhea in Mexico from 2010 to 2014. Patients with underlying gastrointestinal or immunologic diseases, including recent gastrointestinal surgery, were excluded from the study. Stool and blood samples were collected from each child on admission or immediately after admission. Stool samples were collected in two sterile containers, one for the isolation of bacterial pathogens and the other for the detection of rotavirus. The dataset used in this study was obtained from the supplemental table in the accompanying paper [10]. In total, 164 children aged one to 108 months diagnosed with gastroenteritis were included, with 112 subjects having bacterial AG and 52 controls having viral AG. The primary objective of the study was to evaluate the performance of the ML classifier.

Data preprocessing

The obtained dataset contained 19 features, including age, WHO score, sex, height, weight, white blood cells (WBCs), neutrophil, lymphocyte, monocyte, eosinophil, basophil, red blood cells (RBCs), platelet, highest temperature, duration days, days past onset, and rotavirus vaccine doses. Additionally, one outcome variable, the infection group, was extracted. The infection group consisted of 52 cases of rotavirus, 42 cases of *E. coli*, 36 cases of Shigella, and 34 cases of Salmonella. We classified the rotavirus as viral AG, and the rest were categorized as bacterial AG. We also performed two additional feature engineering: neutrophil-lymphocyte ratio (NLR) and platelet-lymphocyte ratio (PLR).

Model development

We chose to use a decision tree classifier because we were also looking to create tree diagrams. For the decision tree model, max_depth was set to 3, random_state to 42, and class_weight to 'balanced' due to the imbalanced dataset. The parameter max_depth represents the depth of the tree. As mentioned earlier, attributes with missing values in the dataset were imputed using IterativeImputer [11].

Model evaluation and validation

Using a cross-validation approach, 80% of the dataset was allocated for training, and the remaining 20% was used for testing. During this process, stratification was performed using the objective variable as an attribute. To ensure consistency, the data were shuffled, and a random seed of 42 was set. The performance evaluation of the models was conducted using various metrics, including accuracy, precision, recall, and F1-score. Additionally, we plotted receiver operating characteristic (ROC) curves for each classifier and calculated the area under the ROC curve (AUC) to assess the models' performance.

Feature importance by the least absolute shrinkage and selection operator (LASSO)

For modeling purposes, attributes with missing values in the dataset were imputed using IterativeImputer [11]. Subsequently, the data were standardized using StandardScaler [12] and normalized using PowerTransformer [13]. To split the data into training and testing sets, a random_state of 42 was set, and 80% of the dataset was allocated for training, while the remaining 20% was used for testing. Feature selection was conducted on this dataset using the least absolute shrinkage and selection operator (LASSO) [14]. In LASSO, lambda represents the parameter of the regularization term [14]. The feature selection process was performed with lambda values ranging from 0.05 to 0.3. Utilizing the selected results, the five-fold cross-validation (CV) was re-evaluated with the decision tree classifier.

Swarmplot of test values for each type of infection including healthy control (HC)

The dataset used in this study also included data from healthy controls (HCs). We utilized this data to compare test values, including those from healthy controls, to assess whether the selected features were specific for bacterial AG.

Tree diagram of the decision tree classifier used in this analysis

ML encounters the so-called black box problem [15], where the process of how the machine selects the target variables remains unclear. However, one advantage of decision tree classifiers is that they are based on a single tree, which allows for the representation of the tree diagram used in this analysis. Nonetheless, a drawback is that their performance might be inferior to decision tree ensemble classifiers. To visualize the tree diagram of the decision tree classifier in this study, we utilized dtreeviz [16].

Statistical analysis

The study utilized the Python programming language, specifically version 3.7.12 (Python Software Foundation, Wilmington, DE). A Mann-Whitney U-test was performed for all continuous variables, while a chi-square test was used for categorical variables. To compare laboratory values in the three groups for each infection, including HCs, Kruskal-Wallis analysis was employed. For multiple comparisons, the Dwass, Steel, and Critchlow-Fligner multiple comparison analysis method was used.

## Results

Study participants

Out of the 164 participants, 63 (38%) were girls and 101 (62%) were boys. All subjects had a median (interquartile range) age of 22.5 (10.0-36.0) months and a median maximum temperature of 38.0 (37.0-38.6) degrees Celsius. There was a significant difference in age between cases and controls (p<0.05), but temperature showed no significance (p = 0.91) (Table 1). Significant differences were found between bacterial and viral AG in all blood count findings, except for neutrophils. No significant differences were found in any of the categorical variables (Table 1).

**Table 1:** Characteristics of this study. All values are pre-imputation values. Continuous variables are reported as median (interquartile range). Categorical variables are reported as percentages, with proportions in parentheses. NLR: neutrophil-lymphocyte ratio, PLR: platelet-lymphocyte ratio.

	Viral AG (N = 52)	Bacterial AG (N = 112)	p-value
Continuous variables			
Age (months)	29.0 (12.8-51.0)	19.0 (8.0-30.3)	<0.05
Height (m)	0.87 (0.73-1.04)	0.80 (0.70-0.92)	0.12
Weight (kg)	12.5 (9.5-17.1)	10.5 (7.5-13.0)	<0.05
WBC (10^6^/L)	8500 (6775-11825)	11975 (8900-15008)	<0.05
Neutrophils (10^6^/L)	5550 (3150-8525)	5750 (3270-8988)	0.48
Lymphocyte (10^6^/L)	2000 (1000-3475)	3395 (1978-5855)	<0.05
Monocyte (10^6^/L)	800 (675-1000)	1035 (595-1425)	<0.05
Eosinophils (10^6^/L)	0.0 (0-0)	60.0 (0-200)	<0.05
Basophil (10^6^/L)	0.0 (0-100)	35.0 (0-105)	<0.05
RBC (10^6^/µL)	4.23 (3.92-4.50)	4.14 (3.77-4.47)	0.16
Platelet (cells/µL)	282500 (243750-349250)	322000 (240250-385000)	0.54
Highest temperature (℃)	38.0 (37.0-38.5)	38.0 (36.9-39.0)	0.91
NLR	2.47 (1.04-8.41)	1.48 (0.75-3.48)	<0.05
PLR	159.51 (79.75-290.09)	87.48 (56.66-30.32)	<0.05
Categorical variables			
WHO_score			0.12
Mild	33 (63%)	62 (55%)	
Moderate	8 (15%)	10 (9%)	
Severe	11 (21%)	40 (36%)	
Sex			0.61
Female	18 (35%)	45 (40%)	
Male	34 (65%)	67 (60%)	
Duration (days)			0.74
0-5	21 (40%)	50 (45%)	
6-10	27 (52%)	50 (45%)	
11-	4 (8%)	10 (9%)	
Onset (days)			0.64
1-3	37 (71%)	74 (66%)	
4-	15 (29%)	38 (34%)	
Vaccine (times)			0.14
0	27 (52%)	43 (38%)	
1-3	25 (48%)	69 (62%)	

Model performance

**Figure 1:**
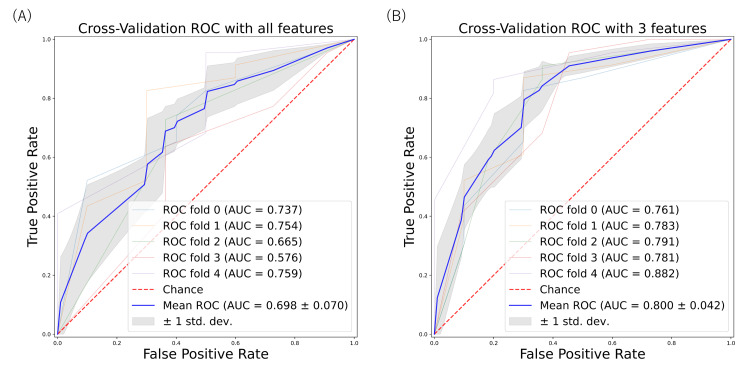
ROC curve for predicting bacterial gastroenterocolitis based on machine learning models. The area under the ROC curve is calculated by ROC curves using all variables (A) and three variables (PLR, eosinophils, and WBC) (B) based on the decision tree classifier. ROC: receiver operating characteristics; PLR: platelet-lymphocyte ratio; WBC: white blood cells; ROC: receiver operating characteristics; AUC: area under ROC curves.

Table 2 shows the results of the classifier, including accuracy, precision (positive predictive value), recall (sensitivity), specificity, F1 score, negative predictive value, area under ROC curves (AUC) using the predict argument, AUC using the predict_proba argument, and area under the precision-recall curve. The predict argument returns the predicted value, while the predict_proba argument returns the probabilities of the predicted values. For AUC, the default classifier with all features demonstrated performance ranging from 0.58 to 0.76, with the mean AUC being 0.70 ± 0.07 (Figure 1).

**Table 2:** Average model performances of five-fold cross-validation. *Three features: platelet-lymphocyte ratio, eosinophils, and white blood cells. PPV: positive predictive value; NPV: negative predictive value; ROC: receiver operating characteristics; AUC: area under ROC curves; PRAUC: area under the precision-recall curve.

Average model performances		
	Scores with all features	Scores with three features*
Accuracy	0.726	0.744
Precision (PPV)	0.812	0.853
Recall (sensitivity)	0.777	0.760
Specificity	0.615	0.713
F1 score	0.791	0.797
NPV	0.595	0.612
AUC (predict)	0.696	0.736
AUC (predict_proba)	0.698	0.800
PRAUC	0.870	0.889

Feature importance

**Figure 2:**
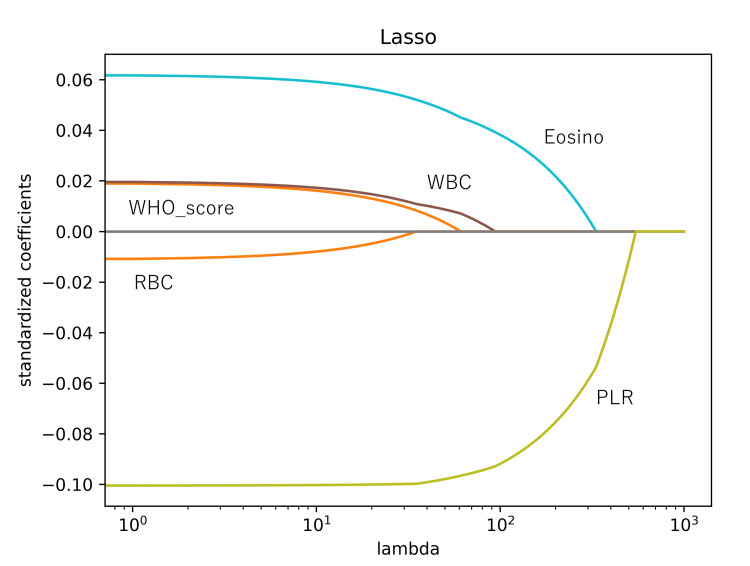
LASSO coefficients as a function of lambda. Lambda represents a parameter in the regularization term. As lambda becomes very large, the LASSO yields the null model, where all coefficient estimates become zero. When moving from left to right in our plot, we observe that the LASSO models initially include many predictors with high magnitudes of coefficient estimates. However, with increasing lambda, the coefficient estimates gradually approach zero. PLR: platelet-lymphocyte ratio; Eosino: eosinophils; WBC: white blood cells; RBC: red blood cells; LASSO: least absolute shrinkage and selection operator.

As lambda increased, the coefficient estimates approached zero, leading to the selection of PLR as the most important feature. The subsequent three feature selections, including eosinophils and white blood cells (WBCs), were determined based on this process (Figure 2). The classifier, using three features (PLR, eosinophil, and WBC), exhibited moderate performance, ranging from 0.76 to 0.88, with the mean AUC being 0.80 ± 0.04 (Figure 1) (Table 2).

Swarmplot of test values for each type of infection including healthy control (HC)

**Figure 3:**
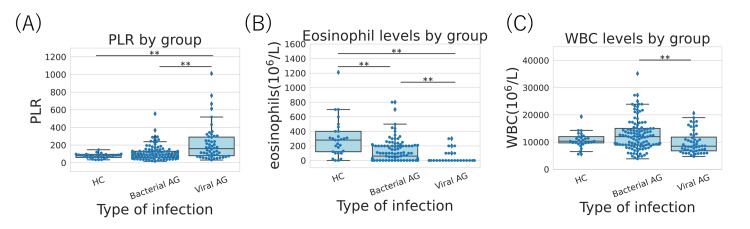
Swarmplot of test values for each type of infection including HC. (A) PLR, (B) eosinophils, and (C) WBC. HC: healthy control; PLR: platelet-lymphocyte ratio; WBC: white blood cells; AG: acute gastroenteritis. **P<0.01.

All three features were found to be significantly different between bacterial and viral AG. Additionally, only eosinophil counts showed a significant difference between HC and bacterial AG (Figure 3).

Tree diagram of the decision tree classifier used in this analysis

**Figure 4:**
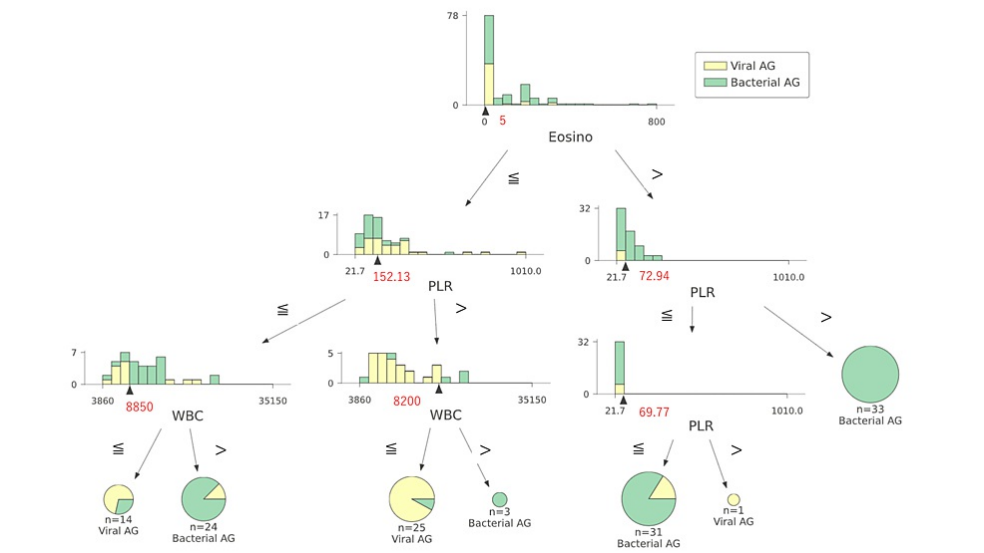
Tree diagram of the decision tree classifier used in this analysis. The red numbers are the thresholds for conditional branching in the decision tree diagram. PLR: platelet-lymphocyte ratio; Eosino: eosinophils; WBC: white blood cells; AG: acute gastroenterocolitis.

According to the tree diagram, the decision tree classifier used in this study initially split the target based on eosinophil count (5 × 10^6^/L) (Figure 4). If the eosinophil count was greater than five, the next conditional branching between bacterial and viral AG was determined by the PLR value. On the other hand, if the eosinophil count was less than five, the subject was further conditionally branched by PLR = 152.13, and finally, bacterial and viral AG were distinguished based on the value of WBC. This tree diagram can be effectively utilized in actual clinical practice, and the results of this analysis should prove valuable for identifying bacterial AG.

## Discussion

This study shows that blood cell counts can be used to narrow down bacterial AG in children with gastrointestinal symptoms with moderate accuracy. PLR, eosinophils, and WBC were important in the feature selection that made this possible. In the following sections, we discuss our findings with reference to the limited literature.

In the past, attempts have been made to identify serum markers capable of differentiating bacterial from viral origins in pediatric acute diarrhea. However, it has been reported that serum markers alone are not sufficient to differentiate between viral and bacterial gastroenteritis in emergency cases [17]. Studies have indicated that procalcitonin values of 1 μg/L or higher show better specificity, sensitivity, and predictive value for distinguishing viral from bacterial infections in children compared to CRP, interleukin 6, or interferon-alpha [18]. Nevertheless, differentiating between viral and bacterial AG based solely on clinical symptoms remains challenging [2].

In our study, we found that PLR, eosinophil counts, and WBC counts were important features in differentiating between bacterial and viral AG. PLR is an inexpensive and easily calculable index that correlates with the prognosis of systemic inflammatory diseases, reflecting inflammation, atherosclerosis, and platelet activation [19]. Eosinophils, on the other hand, are granulocytes that are mostly linked to TH2 reactions to parasites and hyperimmune reactive states like asthma, allergies, and eosinophilic esophagitis. They have also been recognized as regulators of immune homeostasis, suppressing overactive inflammatory responses by secreting specific molecules that attenuate the immune response [20]. WBC counts have long been utilized in the diagnosis of infectious diseases. During bacterial infections, large numbers of neutrophils are consumed. Dynamic changes occur in WBC counts and left-shift data from the onset of infection to recovery, reflecting the severity of the bacterial infection [21]. In our study, we did not find significant differences in neutrophil counts between the two groups, suggesting that neutrophils, being highly variable in production and consumption, may not be sufficient to distinguish between bacterial and viral acute gastroenteritis. Therefore, further evaluation of leukocyte fractions is essential and will be the focus of future studies.

While many machine learning classifiers have black boxes, the decision tree classifier is an attractive model when semantic interpretability is a consideration [22]. In particular, a single decision tree is a weak learner, and overlearning is often experienced as a problem. While tree-based ensemble classifiers, such as bagging and boosting, are preferred for performance, a single decision tree classifier may be an option when semantic interpretability is a consideration. Depending on the dataset, a relatively shallow depth, as in the present case where max_depth = 3, may prevent overlearning and provide the usefulness of semantic interpretability. A decision tree flowchart is a visual map showing clear decision-making pathways. It shows the potential outcomes of various solutions through a network of one-way branches.

One of the significant strengths of this study is its demonstration of the potential of ML algorithms in identifying bacterial AG from blood counts and interviews. By further combining clinical findings and biochemical test results, a more accurate classification method could be achieved. The decision tree classifier, selected in this study, not only represents tree diagrams but also exhibited moderate or better classification ability. This suggests that tree diagrams have practical applications in the real world, and we were able to demonstrate one such indicator. Additionally, this study offers a non-invasive perspective by leveraging previously published data, thereby enhancing its relevance and applicability.

However, there are several limitations that need to be acknowledged in this study. Firstly, the number of infections included is limited, as it only considers rotavirus enteritis in the viral AG. Additionally, bacterial AGs do not include Campylobacter, which is also a common pathogen. These limitations suggest that the results obtained may not fully represent the entire spectrum of pediatric enteritis. Moreover, the decision tree classifier used in this study did not exhibit relatively high classification performance, and the use of an ensemble learner might potentially improve performance. However, we did not compare these two classifiers in this study. Another issue is the adequacy of hyperparameters and feature creation, which could impact the overall accuracy and predictive power of the model. Furthermore, the number of data points in this study is relatively small, and further validation in a larger study cohort would be necessary to strengthen the findings and generalize the results to a broader population.

## Conclusions

In conclusion, this study successfully demonstrated the utility of ML models in predicting bacterial AG using commonly recorded blood counts, achieving moderate performance. By employing a decision tree classifier, a tree diagram was created, presenting a practical flowchart that can aid in clinical decision-making. These results offer valuable insights to narrow down the target population for bacterial AG among numerous hospital visitors. Notably, PLR emerged as a significant finding worthy of attention.

For future work, we plan to incorporate new features, such as clinical and biochemical findings, to create a more powerful classifier with improved accuracy. Additionally, adjustments to the classifier will be explored to enhance its performance further.
